# 2-Hydr­oxy-5-nitro­benzaldehyde 2,4-dinitro­phenyl­hydrazone

**DOI:** 10.1107/S1600536808005825

**Published:** 2008-03-07

**Authors:** Abdassalam A. Tameem, Bahruddin Saad, Abdussalam M. Salhin, Samuel Robinson Jebas, Hoong-Kun Fun

**Affiliations:** a School of Chemical Sciences, Universiti Sains Malaysia, 11800 USM, Penang, Malaysia; b X-ray Crystallography unit, School of Physics, Universiti Sains Malaysia, 11800 USM, Penang, Malaysia

## Abstract

In the title compound, C_13_H_9_N_5_O_7_, one of the nitro groups is twisted away from the attached benzene ring by 16.21 (8)°. The dihedral angle between the two benzene rings is 4.63 (1)°. The mol­ecular structure is stabilized by intra­molecular N—H⋯O and O—H⋯N hydrogen bonds which generate an *S*(6) ring motif. The mol­ecules pack as layers parallel to the *ab* plane; mol­ecules of adjacent layers are linked into chains along the [101] direction through N—H⋯O hydrogen bonds.

## Related literature

For related literature, see: Cordis *et al.* (1998 ▶); Fun *et al.* (1996 ▶); Guillaumont & Nakamura (2000 ▶); Hanoune *et al.* (2006 ▶); Lamberton *et al.* (1974 ▶); Niknam *et al.* (2005 ▶); Raj & Kurup (2007 ▶); Salhin *et al.* (2007 ▶); Shan, Xu *et al.* (2003 ▶); Shan, Yu *et al.* (2003 ▶); Tameem *et al.* (2007 ▶); Uchiyama *et al.* (2003 ▶); Vogel *et al.* (2000 ▶); Zegota (1999 ▶); Zlotorzynska & Lai (1999 ▶). For ring motifs, see: Bernstein *et al.* (1995 ▶).
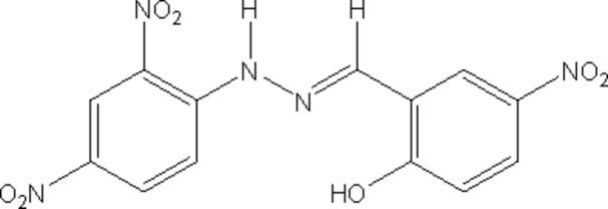


## Experimental

### 

#### Crystal data

C_13_H_9_N_5_O_7_
*M* *_r_* = 347.25
Monoclinic, 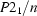

*a* = 12.7543 (5) Å
*b* = 8.1898 (3) Å
*c* = 13.8618 (5) Å
β = 112.683 (2)°
*V* = 1335.94 (9) Å^3^
*Z* = 4
Mo *K*α radiation
μ = 0.14 mm^−1^
*T* = 100.0 (1) K
0.29 × 0.27 × 0.11 mm

#### Data collection

Bruker SMART APEXII CCD area-detector diffractometer
Absorption correction: multi-scan (*SADABS*; Bruker, 2005 ▶) *T*_min_ = 0.960, *T*_max_ = 0.985
24539 measured reflections
5195 independent reflections
3513 reflections with *I* > 2σ(*I*)
*R*_int_ = 0.056

#### Refinement

*R*[*F* ^2^ > 2σ(*F* ^2^)] = 0.050
*wR*(*F* ^2^) = 0.145
*S* = 1.09
5195 reflections
234 parameters
H atoms treated by a mixture of independent and constrained refinement
Δρ_max_ = 0.47 e Å^−3^
Δρ_min_ = −0.33 e Å^−3^

### 

Data collection: *APEX2* (Bruker, 2005 ▶); cell refinement: *APEX2*; data reduction: *SAINT* (Bruker, 2005 ▶); program(s) used to solve structure: *SHELXTL* (Sheldrick, 2008 ▶); program(s) used to refine structure: *SHELXTL*; molecular graphics: *SHELXTL*; software used to prepare material for publication: *SHELXTL* and *PLATON* (Spek, 2003 ▶).

## Supplementary Material

Crystal structure: contains datablocks global, I. DOI: 10.1107/S1600536808005825/ci2567sup1.cif

Structure factors: contains datablocks I. DOI: 10.1107/S1600536808005825/ci2567Isup2.hkl

Additional supplementary materials:  crystallographic information; 3D view; checkCIF report

## Figures and Tables

**Table 1:** Hydrogen-bond geometry (Å, °)

*D*—H⋯*A*	*D*—H	H⋯*A*	*D*⋯*A*	*D*—H⋯*A*
N1—H1*N*1⋯O4^i^	0.89 (2)	2.54 (2)	3.0666 (15)	118 (2)
N1—H1*N*1⋯O1	0.89 (2)	2.07 (2)	2.6477 (15)	121 (2)
O5—H1*O*5⋯N2	0.92 (3)	1.82 (3)	2.6656 (14)	150 (2)

Symmetry code: (i) 
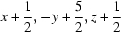
.

**Fig. 1.**
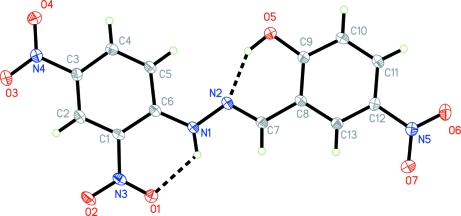
The molecular structure of the title compound, showing 50% probability displacement ellipsoids and the atomic numbering scheme. Hydrogen bonds are shown as dashed lines.

**Fig. 2.**
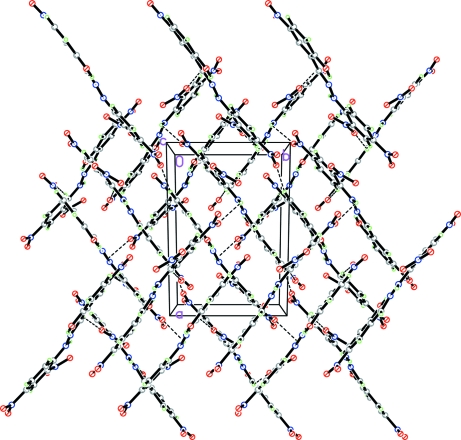
The crystal packing of the title compound, viewed along the c axis. Hydrogen bonds are shown as dashed lines.

## supplementary crystallographic information

### Comment

Phenylhydrazone derivatives have been synthesized in order to investigate their structures and analytical applications. 2,4-Dinitrophenylhydrazones play an important role as stabilizers for the detection, characterization and protection of the carbonyl group of compounds than phenylhydrazones (Niknam *et al.*, 2005). 2,4-Dinitrophenylhydrazone derivatives are widely used in various forms of analytical chemistry (Lamberton *et al.*, 1974; Zegota, 1999; Cordis *et al.*, 1998; Zlotorzynska & Lai, 1999) and are also used as dyes (Guillaumont & Nakamura, 2000). They are also found to have versatile coordinating abilities towards different metal ions (Raj *et al.*, 2006). In addition, they are used to determine airborne aldehydes and ketones (Vogel *et al.*, 2000) and as detectors of formaldehyde (Hanoune *et al.*, 2006). These compounds can exist as E and *Z* stereoisomers (Uchiyama *et al.*, 2003). Their existence as a keto tautomer in the solid state with an E configuration across the C—N bond have been reported (Fun *et al.*, 1996). The title compound, whose structure is reported here, is one of the series of phenylhydrazone derivatives that we have prepared; the crystal structures of some of these compounds have been studied previously (Tameem *et al.*, 2007; Salhin *et al.*, 2007).

The bond lengths and angles in the title compound (Fig.1) have normal values. The molecule is nealy planar, with a maximum deviation from the mean plane of 0.487 (1) Å for atom O1. The dihedral angle between the two benzene rings is 4.63 (1)°. The C—N bond lengths in the hydrazone moiety agree well with those reported earlier (Shan, Xu *et al.*, 2003; Shan, Yu *et al.*, 2003). The asymmetry of the exocyclic angles at C7 [C7—C8—C13 = 118.2 (2)° and C7—C8—C9 = 123.3 (2)°] is more pronounced than that at C6 [N1—C6—C1 = 122.7 (1)° and N1—C6—C5 = 120.5 (1)°]. One of the hydrazone N atoms is involved in an O—H···N intramolecular hydrogen bond with the hydroxy group, while the other is involved in an N—H···O intramolecular hydrogen bond with the nitro group. Each of these hydrogen bonds generate an S(6) ring motif (Bernstein *et al.*,1995).

The molecules are linked into a chain along the [1 0 1] direction through N—H—O hydrogen bonds. The molecules pack as layers parallel to the *ab* plane. Within the layer, weak π-π interactions are observed between the C1—C6 and C8—C13 benzene rings, with a centroid-centroid distance of 3.7457 (8) Å.

### Experimental

2,4-Dinitrophenylhydrazine (400 mg, 2 mmol) in concentrated sulfuric acid (5 ml) was slowly added to a solution of 2-hydroxy-5-nitrobenzaldehyde (337 mg, 2 mmol) in ethanol (95%, 20 ml). The mixture was stirred for 15 min, and was left to stand at room temperature for 30 min. The resulting product was filtered and washed with 95% ethanol (20 ml) and the orange powder product was collected. Crystals suitable for X-ray diffraction analysis were grown by slow evaporation of a saturated solution of the resulted compound in ethanol.

### Refinement

O– and N-bound H atoms were located in a difference map and refined isotropically. The remaining H atoms were placed in calculated positions (C—H = 0.93 Å) and refined using a riding model, with *U*_iso_(H) = 1.2*U*_eq_(C).

### Crystal data

**Table:**

C_13_H_9_N_5_O_7_	*F*_000_ = 712
*M**_r_* = 347.25	*D*_x_ = 1.726 Mg m^−^^3^
Monoclinic, *P*2_1_/*n*	Mo *K*α radiation λ = 0.71073 Å
Hall symbol: -P 2yn	Cell parameters from 3255 reflections
*a* = 12.7543 (5) Å	θ = 2.8–33.1º
*b* = 8.1898 (3) Å	µ = 0.14 mm^−^^1^
*c* = 13.8618 (5) Å	*T* = 100.0 (1) K
β = 112.683 (2)º	Block, orange
*V* = 1335.94 (9) Å^3^	0.29 × 0.27 × 0.11 mm
*Z* = 4	

### Data collection

**Table:**

Bruker SMART APEXII CCD area-detector diffractometer	3513 reflections with *I* > 2σ(*I*)
Detector resolution: 8.33 pixels mm^-1^	*R*_int_ = 0.056
ω scans	θ_max_ = 33.4º
Absorption correction: multi-scan(SADABS; Bruker, 2005)	θ_min_ = 2.8º
*T*_min_ = 0.960, *T*_max_ = 0.985	*h* = −19→19
24539 measured reflections	*k* = −10→12
5195 independent reflections	*l* = −21→20

### Refinement

**Table:**

Refinement on *F*^2^	H atoms treated by a mixture of independent and constrained refinement
Least-squares matrix: full	*w* = 1/[σ^2^(*F*_o_^2^) + (0.0708*P*)^2^ + 0.0132*P*] where *P* = (*F*_o_^2^ + 2*F*_c_^2^)/3
*R*[*F*^2^ > 2σ(*F*^2^)] = 0.050	(Δ/σ)_max_ = 0.001
*wR*(*F*^2^) = 0.145	Δρ_max_ = 0.47 e Å^−^^3^
*S* = 1.09	Δρ_min_ = −0.33 e Å^−^^3^
5195 reflections	Extinction correction: none
234 parameters	

### Special details

**Table:**

Geometry. Experimental. The low-temperature data was collected with the Oxford Crysosystem Cobra low-temperature attachement.All e.s.d.'s (except the e.s.d. in the dihedral angle between two l.s. planes) are estimated using the full covariance matrix. The cell e.s.d.'s are taken into account individually in the estimation of e.s.d.'s in distances, angles and torsion angles; correlations between e.s.d.'s in cell parameters are only used when they are defined by crystal symmetry. An approximate (isotropic) treatment of cell e.s.d.'s is used for estimating e.s.d.'s involving l.s. planes.

### Fractional atomic coordinates and isotropic or equivalent isotropic displacement parameters (Å^2^)

**Table:**

	*x*	*y*	*z*	*U*_iso_*/*U*_eq_	
O1	0.71240 (8)	0.91583 (13)	0.22343 (8)	0.0228 (2)	
O2	0.61439 (9)	1.12841 (14)	0.22968 (8)	0.0282 (2)	
O3	0.39947 (10)	1.41385 (15)	−0.08171 (9)	0.0340 (3)	
O4	0.45224 (10)	1.41148 (14)	−0.21219 (8)	0.0307 (3)	
O5	0.94762 (8)	0.81549 (12)	−0.11896 (7)	0.0189 (2)	
O6	1.34597 (9)	0.33913 (14)	0.11735 (8)	0.0300 (3)	
O7	1.29887 (9)	0.41281 (13)	0.24496 (8)	0.0259 (2)	
N1	0.80661 (9)	0.91651 (14)	0.08340 (9)	0.0164 (2)	
N2	0.87737 (9)	0.86266 (13)	0.03680 (8)	0.0157 (2)	
N3	0.66026 (9)	1.04241 (14)	0.18460 (8)	0.0187 (2)	
N4	0.46053 (10)	1.36459 (15)	−0.12555 (9)	0.0208 (2)	
N5	1.28791 (9)	0.41996 (14)	0.15301 (9)	0.0184 (2)	
C1	0.64902 (11)	1.08800 (16)	0.07968 (9)	0.0160 (2)	
C2	0.56488 (11)	1.20121 (16)	0.02874 (10)	0.0179 (2)	
H2A	0.5209	1.2465	0.062	0.021*	
C3	0.54779 (10)	1.24505 (16)	−0.07199 (10)	0.0172 (2)	
C4	0.61284 (11)	1.17695 (16)	−0.12363 (10)	0.0171 (2)	
H4A	0.5993	1.2067	−0.1921	0.02*	
C5	0.69644 (11)	1.06624 (16)	−0.07233 (10)	0.0165 (2)	
H5A	0.7392	1.0211	−0.1069	0.02*	
C6	0.71925 (10)	1.01896 (15)	0.03210 (9)	0.0150 (2)	
C7	0.96469 (10)	0.78013 (15)	0.09632 (9)	0.0151 (2)	
H7A	0.9782	0.7678	0.1668	0.018*	
C8	1.04195 (10)	0.70636 (15)	0.05474 (9)	0.0142 (2)	
C9	1.03109 (10)	0.72528 (15)	−0.05001 (9)	0.0151 (2)	
C10	1.10725 (11)	0.64726 (16)	−0.08556 (10)	0.0168 (2)	
H10A	1.1009	0.6634	−0.154	0.02*	
C11	1.19152 (10)	0.54678 (16)	−0.02006 (10)	0.0169 (2)	
H11A	1.2412	0.4933	−0.044	0.02*	
C12	1.20051 (10)	0.52732 (15)	0.08265 (10)	0.0159 (2)	
C13	1.12875 (10)	0.60635 (15)	0.12052 (10)	0.0153 (2)	
H13A	1.1382	0.593	0.19	0.018*	
H1N1	0.8262 (16)	0.891 (2)	0.1508 (16)	0.038 (5)*	
H1O5	0.905 (2)	0.854 (3)	−0.082 (2)	0.067 (8)*	

### Atomic displacement parameters (Å^2^)

**Table:**

	*U*^11^	*U*^22^	*U*^33^	*U*^12^	*U*^13^	*U*^23^
O1	0.0238 (5)	0.0253 (5)	0.0220 (5)	0.0056 (4)	0.0118 (4)	0.0049 (4)
O2	0.0377 (6)	0.0307 (6)	0.0253 (5)	0.0079 (5)	0.0222 (5)	−0.0004 (4)
O3	0.0317 (6)	0.0461 (7)	0.0298 (6)	0.0207 (5)	0.0181 (5)	0.0077 (5)
O4	0.0379 (6)	0.0342 (6)	0.0228 (5)	0.0153 (5)	0.0150 (5)	0.0087 (4)
O5	0.0201 (4)	0.0210 (5)	0.0162 (4)	0.0047 (4)	0.0076 (3)	0.0023 (3)
O6	0.0284 (5)	0.0335 (6)	0.0285 (5)	0.0145 (5)	0.0114 (4)	0.0014 (4)
O7	0.0269 (5)	0.0307 (6)	0.0197 (5)	0.0070 (4)	0.0086 (4)	0.0059 (4)
N1	0.0163 (5)	0.0196 (5)	0.0155 (5)	0.0030 (4)	0.0085 (4)	0.0005 (4)
N2	0.0158 (5)	0.0157 (5)	0.0177 (5)	0.0000 (4)	0.0089 (4)	−0.0017 (4)
N3	0.0193 (5)	0.0220 (6)	0.0183 (5)	−0.0015 (4)	0.0112 (4)	−0.0006 (4)
N4	0.0209 (5)	0.0224 (6)	0.0204 (5)	0.0049 (4)	0.0097 (4)	0.0004 (4)
N5	0.0170 (5)	0.0177 (5)	0.0203 (5)	0.0010 (4)	0.0070 (4)	0.0005 (4)
C1	0.0177 (5)	0.0183 (6)	0.0144 (5)	−0.0009 (4)	0.0088 (4)	−0.0011 (4)
C2	0.0176 (5)	0.0191 (6)	0.0195 (6)	0.0006 (5)	0.0099 (5)	−0.0033 (5)
C3	0.0161 (5)	0.0174 (6)	0.0194 (6)	0.0029 (5)	0.0081 (5)	0.0005 (4)
C4	0.0194 (6)	0.0174 (6)	0.0158 (5)	−0.0005 (5)	0.0085 (4)	−0.0005 (4)
C5	0.0169 (5)	0.0185 (6)	0.0160 (5)	0.0001 (5)	0.0085 (4)	−0.0023 (4)
C6	0.0147 (5)	0.0150 (6)	0.0163 (5)	−0.0019 (4)	0.0070 (4)	−0.0017 (4)
C7	0.0161 (5)	0.0151 (6)	0.0158 (5)	−0.0009 (4)	0.0081 (4)	−0.0004 (4)
C8	0.0151 (5)	0.0143 (5)	0.0142 (5)	−0.0009 (4)	0.0068 (4)	−0.0013 (4)
C9	0.0156 (5)	0.0142 (6)	0.0156 (5)	−0.0007 (4)	0.0062 (4)	−0.0002 (4)
C10	0.0190 (6)	0.0181 (6)	0.0156 (5)	−0.0011 (5)	0.0093 (4)	−0.0010 (4)
C11	0.0159 (5)	0.0174 (6)	0.0200 (6)	−0.0012 (4)	0.0096 (5)	−0.0024 (4)
C12	0.0134 (5)	0.0140 (6)	0.0190 (6)	0.0010 (4)	0.0048 (4)	0.0005 (4)
C13	0.0160 (5)	0.0147 (6)	0.0161 (5)	−0.0009 (4)	0.0072 (4)	0.0000 (4)

### Geometric parameters (Å, °)

**Table:**

O1—N3	1.2372 (15)	C2—H2A	0.93
O2—N3	1.2291 (15)	C3—C4	1.4035 (18)
O3—N4	1.2262 (15)	C4—C5	1.3711 (18)
O4—N4	1.2259 (15)	C4—H4A	0.93
O5—C9	1.3446 (15)	C5—C6	1.4159 (17)
O5—H1O5	0.92 (3)	C5—H5A	0.93
O6—N5	1.2302 (15)	C7—C8	1.4514 (17)
O7—N5	1.2288 (14)	C7—H7A	0.93
N1—C6	1.3565 (16)	C8—C13	1.3962 (17)
N1—N2	1.3697 (15)	C8—C9	1.4128 (16)
N1—H1N1	0.89 (2)	C9—C10	1.4014 (18)
N2—C7	1.2920 (16)	C10—C11	1.3799 (18)
N3—C1	1.4541 (16)	C10—H10A	0.93
N4—C3	1.4530 (17)	C11—C12	1.3927 (17)
N5—C12	1.4589 (16)	C11—H11A	0.93
C1—C2	1.3876 (18)	C12—C13	1.3795 (17)
C1—C6	1.4187 (17)	C13—H13A	0.93
C2—C3	1.3753 (17)		
			
C9—O5—H1O5	105.4 (15)	C4—C5—H5A	119.2
C6—N1—N2	120.62 (11)	C6—C5—H5A	119.2
C6—N1—H1N1	122.4 (13)	N1—C6—C5	120.57 (11)
N2—N1—H1N1	116.6 (13)	N1—C6—C1	122.75 (11)
C7—N2—N1	115.45 (10)	C5—C6—C1	116.66 (11)
O2—N3—O1	122.71 (11)	N2—C7—C8	120.94 (11)
O2—N3—C1	118.60 (11)	N2—C7—H7A	119.5
O1—N3—C1	118.64 (11)	C8—C7—H7A	119.5
O4—N4—O3	123.45 (12)	C13—C8—C9	118.32 (11)
O4—N4—C3	118.12 (11)	C13—C8—C7	118.27 (11)
O3—N4—C3	118.43 (11)	C9—C8—C7	123.37 (11)
O7—N5—O6	123.08 (11)	O5—C9—C10	117.82 (11)
O7—N5—C12	118.31 (11)	O5—C9—C8	121.85 (11)
O6—N5—C12	118.60 (11)	C10—C9—C8	120.32 (11)
C2—C1—C6	122.19 (11)	C11—C10—C9	120.67 (11)
C2—C1—N3	116.05 (11)	C11—C10—H10A	119.7
C6—C1—N3	121.76 (11)	C9—C10—H10A	119.7
C3—C2—C1	118.67 (12)	C10—C11—C12	118.49 (12)
C3—C2—H2A	120.7	C10—C11—H11A	120.8
C1—C2—H2A	120.7	C12—C11—H11A	120.8
C2—C3—C4	121.38 (12)	C13—C12—C11	122.02 (11)
C2—C3—N4	119.01 (11)	C13—C12—N5	118.45 (11)
C4—C3—N4	119.61 (11)	C11—C12—N5	119.53 (11)
C5—C4—C3	119.51 (12)	C12—C13—C8	120.14 (11)
C5—C4—H4A	120.2	C12—C13—H13A	119.9
C3—C4—H4A	120.2	C8—C13—H13A	119.9
C4—C5—C6	121.55 (12)		
			
C6—N1—N2—C7	172.84 (11)	C2—C1—C6—C5	−2.48 (18)
O2—N3—C1—C2	−15.33 (17)	N3—C1—C6—C5	176.74 (11)
O1—N3—C1—C2	162.33 (11)	N1—N2—C7—C8	175.64 (11)
O2—N3—C1—C6	165.40 (12)	N2—C7—C8—C13	−174.24 (11)
O1—N3—C1—C6	−16.94 (18)	N2—C7—C8—C9	3.12 (19)
C6—C1—C2—C3	1.35 (19)	C13—C8—C9—O5	177.95 (11)
N3—C1—C2—C3	−177.92 (11)	C7—C8—C9—O5	0.58 (19)
C1—C2—C3—C4	0.5 (2)	C13—C8—C9—C10	−1.09 (18)
C1—C2—C3—N4	−179.46 (11)	C7—C8—C9—C10	−178.46 (11)
O4—N4—C3—C2	174.55 (13)	O5—C9—C10—C11	−176.95 (11)
O3—N4—C3—C2	−5.12 (19)	C8—C9—C10—C11	2.12 (19)
O4—N4—C3—C4	−5.37 (19)	C9—C10—C11—C12	−1.25 (19)
O3—N4—C3—C4	174.96 (13)	C10—C11—C12—C13	−0.63 (19)
C2—C3—C4—C5	−1.0 (2)	C10—C11—C12—N5	179.16 (11)
N4—C3—C4—C5	178.90 (11)	O7—N5—C12—C13	−4.77 (17)
C3—C4—C5—C6	−0.22 (19)	O6—N5—C12—C13	174.17 (12)
N2—N1—C6—C5	2.89 (18)	O7—N5—C12—C11	175.44 (12)
N2—N1—C6—C1	−175.04 (11)	O6—N5—C12—C11	−5.62 (18)
C4—C5—C6—N1	−176.16 (12)	C11—C12—C13—C8	1.65 (19)
C4—C5—C6—C1	1.89 (18)	N5—C12—C13—C8	−178.14 (11)
C2—C1—C6—N1	175.52 (12)	C9—C8—C13—C12	−0.75 (18)
N3—C1—C6—N1	−5.25 (19)	C7—C8—C13—C12	176.75 (11)

### Hydrogen-bond geometry (Å, °)

**Table:**

*D*—H···*A*	*D*—H	H···*A*	*D*···*A*	*D*—H···*A*
N1—H1N1···O4^i^	0.89 (2)	2.54 (2)	3.0666 (15)	118 (2)
N1—H1N1···O1	0.89 (2)	2.07 (2)	2.6477 (15)	121 (2)
O5—H1O5···N2	0.92 (3)	1.82 (3)	2.6656 (14)	150 (2)

Symmetry codes: (i) *x*+1/2, −*y*+5/2, *z*+1/2.
